# Suppression of Oncolytic Adenovirus-Mediated Hepatotoxicity by Liver-Specific Inhibition of NF-κB

**DOI:** 10.1016/j.omto.2017.10.003

**Published:** 2017-10-26

**Authors:** Mitsuhiro Machitani, Fuminori Sakurai, Keisaku Wakabayashi, Kosuke Nakatani, Masashi Tachibana, Nobuyuki Kato, Toshiyoshi Fujiwara, Hiroyuki Mizuguchi

**Affiliations:** 1 Laboratory of Biochemistry and Molecular Biology, Graduate School of Pharmaceutical Sciences, Osaka University, 1-6 Yamadaoka, Suita, Osaka 565-0871, Japan; 2 Institute for Frontier Life and Medical Sciences, Kyoto University, 53 Shogoin Kawahara-cho, Sakyo-ku, Kyoto 606-8507, Japan; 3 Laboratory of Regulatory Sciences for Oligonucleotide Therapeutics, Clinical Drug Development Unit, Graduate School of Pharmaceutical Sciences, Osaka University, 1-6 Yamadaoka, Suita, Osaka 565-0871, Japan; 4 Global Center for Medical Engineering and Informatics, Osaka University, 2-2 Yamadaoka, Suita, Osaka 565-0871, Japan; 5 Department of Tumor Virology, Okayama University Graduate School of Medicine, Dentistry, and Pharmaceutical Sciences, 2-5-1 Shikata-cho, Kita-ku, Okayama 700-8558, Japan; 6 Department of Gastroenterological Surgery, Okayama University Graduate School of Medicine, Dentistry, and Pharmaceutical Sciences, 2-5-1 Shikata-cho, Kita-ku, Okayama 700-8558, Japan; 7 Laboratory of Hepatocyte Regulation, National Institutes of Biomedical Innovation, Health and Nutrition, 7-6-8 Saito, Asagi, Ibaraki, Osaka 567-0085, Japan; 8 iPS Cell-Based Research Project on Hepatic Toxicity and Metabolism, Graduate School of Pharmaceutical Sciences, Osaka University, 1-6 Yamadaoka, Suita, Osaka 565-0871, Japan; 9 Graduate School of Medicine, Osaka University, 2-2 Yamadaoka, Suita, Osaka 565-0871, Japan

**Keywords:** oncolytic adenovirus, NF-κB, liver-specific promoter, hepatotoxicity

## Abstract

Telomerase-specific replication-competent adenoviruses (Ads), i.e., TRADs, which possess an E1 gene expression cassette driven by the human telomerase reverse transcriptase promoter, are promising agents for cancer treatment. However, even though oncolytic Ads, including TRAD, are intratumorally administered, they are disseminated from the tumor to systemic circulation, causing concern about oncolytic Ad-mediated hepatotoxicity (due mainly to leaky expression of Ad genes in liver). We reported that inhibition of nuclear factor-κB (NF-κB) leads to the suppression of replication-incompetent Ad vector-mediated hepatotoxicity via reduction of the leaky expression of Ad genes in liver. Here, to develop a TRAD with an improved safety profile, we designed a TRAD that carries a liver-specific promoter-driven dominant-negative IκBα (DNIκBα) expression cassette (TRAD-DNIκBα). Compared with a conventional TRAD, TRAD-DNIκBα showed hepatocyte-specific inhibition of NF-κB signaling and significantly reduced Ad gene expression and replication in the normal human hepatocyte cell line. TRAD-induced hepatotoxicity was largely suppressed in mice following intravenous administration of TRAD-DNIκBα. However, the replication profiles and oncolytic activities of TRAD-DNIκBα were comparable with those of the conventional TRAD in human non-hepatic tumor cells. These results indicate that oncolytic Ads containing the liver-specific DNIκBα expression cassette have improved safety profiles without inhibiting oncolytic activities.

## Introduction

Oncolytic viruses, which can preferentially replicate in tumor cells and induce tumor regression, have been actively pursued as potential agents for tumor treatment. Several clinical trials using oncolytic viruses have been carried out, and promising results have been reported.^1^, ^2^ For example, a herpesvirus-based oncolytic virus called T-VEC^3^ was recently approved for cancer treatment by a US Food and Drug Administration (FDA) committee. An adenovirus (Ad) is another promising framework as an oncolytic virus. Various types of oncolytic Ads have been developed using genetic engineering to achieve tumor cell-specific replication and superior antitumor effects.^4^ The telomerase-specific replication-competent Ad (TRAD), which carries the human telomerase reverse transcriptase (hTERT) promoter-driven E1 gene expression cassette, is one of the most promising oncolytic Ads.^5^, ^6^ TRAD shows efficient tumor-specific replication, because most tumor cells highly express telomerase but normal cells do not. A phase I clinical trial using TRAD has already been completed, and TRAD exhibited potent antitumor effects.^7^

Currently, almost all of the clinical applications of oncolytic Ads are limited to local administration into tumor regions.^4^ Even though oncolytic Ads are intratumorally administered, they are disseminated from the tumor to the systemic circulation. The disseminated oncolytic Ads rapidly accumulate in the liver because of the strong hepatotropism of Ads.^8^ Several groups, including ours, have reported that after the accumulation of recombinant Ads (including oncolytic Ads) in the liver, Ad gene expression was seen in the liver, leading to hepatotoxicity.^9^, ^10^, ^11^, ^12^, ^13^, ^14^ In early clinical trials, the intra-arterial administration of oncolytic Ads indeed induced an increase in serum biomarkers of hepatotoxicity.^15^, ^16^ The development of a system that prevents oncolytic Ad-mediated hepatotoxicity would lead to safer cancer virotherapies that use oncolytic Ads.

We recently demonstrated that nuclear factor-κB (NF-κB) promotes not only the leaky expression of Ad genes following transduction with a replication-incompetent Ad vector, but also Ad gene expression following infection with a wild-type Ad.^17^ NF-κB is a ubiquitous transcriptional factor that promotes the expression of a large number of genes, particularly gene families associated with host immune responses.^18^ NF-κB also plays a crucial role in the expression of numerous viral genes.^18^ Under normal conditions, NF-κB stays in the cytoplasm via association with an NF-κB-inhibitory factor, IκBα. Upon stimulation with cytokines and pathogens, IκBα is phosphorylated and degraded, leading to the translocation of NF-κB from the cytoplasm to the nucleus and the expression of the target genes.

In our earlier study mentioned above, we constructed a replication-incompetent Ad vector expressing a dominant-negative IκBα (DNIκBα), which is a negative regulator of NF-κB.^17^ An Ad vector expressing DNIκBα showed the significant inhibition of NF-κB signaling and suppressed the NF-κB-mediated leaky expression of Ad genes, leading to a significant suppression of Ad vector-mediated hepatotoxicity following systemic administration. These results led us to hypothesize that TRAD-mediated hepatotoxicity is also circumvented by suppressing the Ad gene expression in the liver via the liver-specific expression of DNIκBα.

In the present study, we developed a TRAD showing a liver-specific expression of DNIκBα (TRAD-DNIκBα). Compared to the conventional TRAD, TRAD-DNIκBα exhibited significantly lower levels of Ad gene expression and Ad replication in a normal human hepatocyte cell line. In addition, the expression of DNIκBα significantly reduced TRAD-mediated hepatotoxicity. TRAD-DNIκBα exhibited tumor-cell-killing activities comparable with those of the conventional TRAD in human non-hepatic tumor cells.

## Results

### Suppression of Viral Gene Expression and Replication of TRAD by the Inhibition of NF-κB

We previously demonstrated that NF-κB promotes a leaky expression of Ad genes following transduction with recombinant Ads.^17^ In addition, NF-κB binds to an hTERT promoter.^19^ To determine whether NF-κB enhances Ad gene expression and the replication of TRAD in hepatocytes, in the present study we pretreated PH5CH8 cells^20^ (a non-neoplastic human hepatocyte cell line) with an NF-κB inhibitor BAY11-7082 and a proteasome inhibitor MG-132, followed by infection with the conventional TRAD. PH5CH8 cells have often been used as a model of normal human hepatocytes.^20^, ^21^, ^22^ MG-132 is often used as an NF-κB inhibitor because MG-132 inhibits the proteasome-mediated degradation of IκBα.^23^

**Figure 1:**
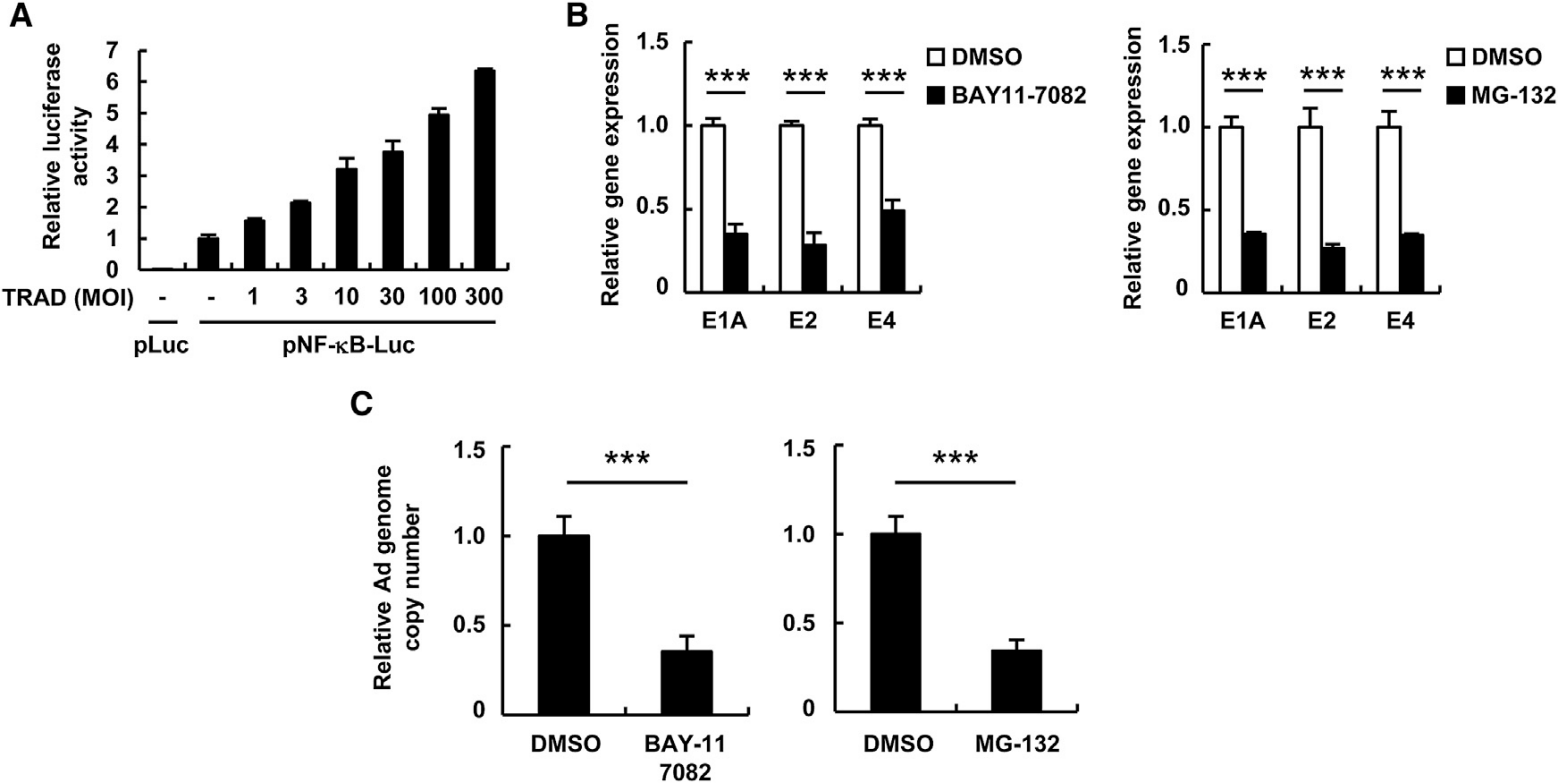
Suppression of Ad Gene Expression and TRAD Replication by NF-κB Inhibitors (A) PH5CH8 cells were transfected with a control plasmid (pLuc) or pNF-κB-Luc, followed by infection with the conventional TRAD at the indicated MOIs. After 24-hr incubation, luciferase activity was determined. The data show firefly luciferase (FLuc) activity normalized by renilla luciferase (RLuc) activity in the cells. (B and C) PH5CH8 cells were pretreated with BAY11-7082 and MG-132 at 5 and 2.5 μM, respectively, for 1 hr, followed by infection with the conventional TRAD at an MOI of 5. After 24-hr incubation, the E1A, E2, and E4 mRNA levels (B) and the copy numbers of TRAD genomic DNA (C) in the cells were determined by qRT-PCR and qPCR analyses, respectively. Data are the means ± SD (n = 3–4). ***p < 0.001.

We observed that the hTERT mRNA levels in PH5CH8 cells were much lower than those in the hepatic tumor cell lines, i.e., the HuH7 and HepG2 cells (Figure S1). The viability of the PH5CH8 cells was not significantly reduced following treatment with BAY11-7082 or MG-132 alone (Figure S2). BAY11-7082 and MG-132 efficiently inhibited NF-κB signaling in PH5CH8 cells (Figure S3). Infection with the conventional TRAD led to a significant activation of NF-κB signaling in PH5CH8 cells in a dose-dependent manner (Figure 1A). The inhibition of NF-κB signaling by BAY11-7082 and MG-132 resulted in a >50% reduction in the E1A, E2, and E4 gene expression in PH5CH8 cells following infection with the conventional TRAD (Figure 1B). Pretreatment with BAY11-7082 and MG-132 reduced the copy numbers of the conventional TRAD genome by >50% in PH5CH8 cells (Figure 1C). These results indicated that the undesired Ad gene expression and replication of TRAD in normal hepatocytes can be suppressed by the inhibition of NF-κB.

### Development of an Oncolytic Ad Expressing a DNIκBα

**Figure 2:**
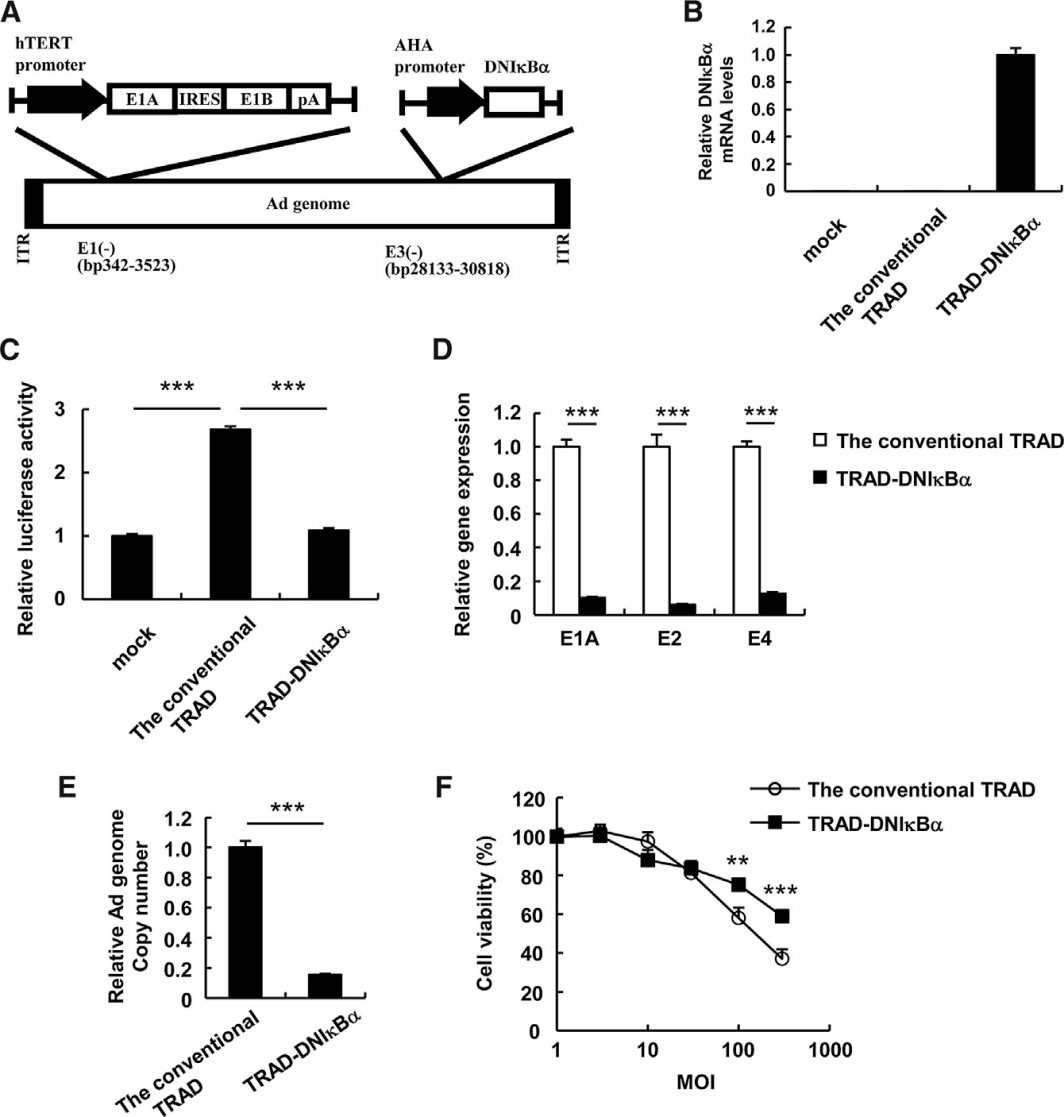
Suppression of Ad Gene Expression and TRAD Replication by the Expression of a Dominant-Negative IκBα in Hepatocytes (A) A schematic diagram of TRAD showing a liver-specific expression of DNκBα (TRAD-DNIκBα). (B) PH5CH8 cells were infected with the conventional TRAD or TRAD-DNIκBα at an MOI of 5. After 48-hr incubation, the DNIκBα mRNA levels were determined by qRT-PCR analysis. (C) PH5CH8 cells were transfected with pNF-κB-Luc, followed by infection with the conventional TRAD or TRAD-DNIκBα at an MOI of 5. After 48-hr incubation, luciferase activity was determined. The data show FLuc activity normalized by RLuc activity in the cells. (D and E) PH5CH8 cells were infected with the conventional TRAD or TRAD-DNIκBα at an MOI of 5. After 48-hr incubation, the E1A, E2, and E4 mRNA levels (D) and the copy numbers of TRAD genomic DNA (E) in the cells were determined by qRT-PCR and qPCR analyses, respectively. (F) PH5CH8 cells were infected with the conventional TRAD or TRAD-DNIκBα at the indicated MOI. After 5-day incubation, cell viability was determined by alamarBlue assay. The data were normalized by the data of the mock-infected group. Data are the means ± SD (n = 3–4). **p < 0.01; ***p < 0.001. AHA promoter, a liver-specific apolipoprotein E enhancer-hepatocyte control region-human a1-antitripsin promoter; DNIκBα, a dominant-negative IκBα; hTERT promoter, a human telomerase reverse transcriptase promoter; IRES, internal ribosome entry site; pA, bovine growth hormone (BGH) poly-adenine sequence.

Next, to suppress the undesired viral gene expression and TRAD replication in the hepatocytes by inhibiting NF-κB signaling, we constructed a TRAD showing a liver-specific expression of DNκBα (TRAD-DNIκBα), which is a negative regulator of NF-κB^24^ (Figure 2A). The expression of DNIκBα is driven by a hepatocyte-specific AHA promoter (a synthetic liver-specific promoter composed of apolipoprotein E enhancer, the hepatocyte control region, and human α1-antitrypsin promoter^25^) to achieve hepatocyte-specific inhibition of NF-κB signaling. An AHA promoter mediates transcription that is highly specific for hepatocytes.^25^, ^26^, ^27^

Here, TRAD-DNIκBα efficiently expressed DNIκBα in PH5CH8 cells (Figure 2B). Infection with the conventional TRAD led to a significant activation of NF-κB signaling in PH5CH8 cells, whereas the activation of NF-κB signaling was significantly suppressed following infection with TRAD-DNIκBα compared with the conventional TRAD (Figure 2C).

We next determined the Ad gene mRNA levels and the copy numbers of TRAD genome in PH5CH8 cells following infection with the conventional TRAD or TRAD-DNIκBα at an MOI of 5. The E1A, E2, and E4 gene expression levels after infection with TRAD-DNIκBα were significantly lower (by >80%) compared with those in the conventional TRAD-infected cells (Figure 2D). Next, we examined the replication efficiencies and cytotoxicity of TRAD-DNIκBα in the hepatocytes. We observed a >80% reduction in the copy numbers of the TRAD genome for TRAD-DNIκBα compared with the conventional TRAD (Figure 2E). TRAD-DNIκBα showed significantly lower cytotoxicity in PH5CH8 cells compared with the conventional TRAD, when infected at an MOI of more than 100 (Figure 2F). These results indicate that TRAD-DNIκBα exhibits a significant reduction in undesired viral gene expression and replication in the hepatocytes by overexpressing DNIκBα in a hepatocyte-specific manner.

### Suppression of TRAD-Induced Hepatotoxicity by DNIκBα Expression in Mice

**Figure 3:**
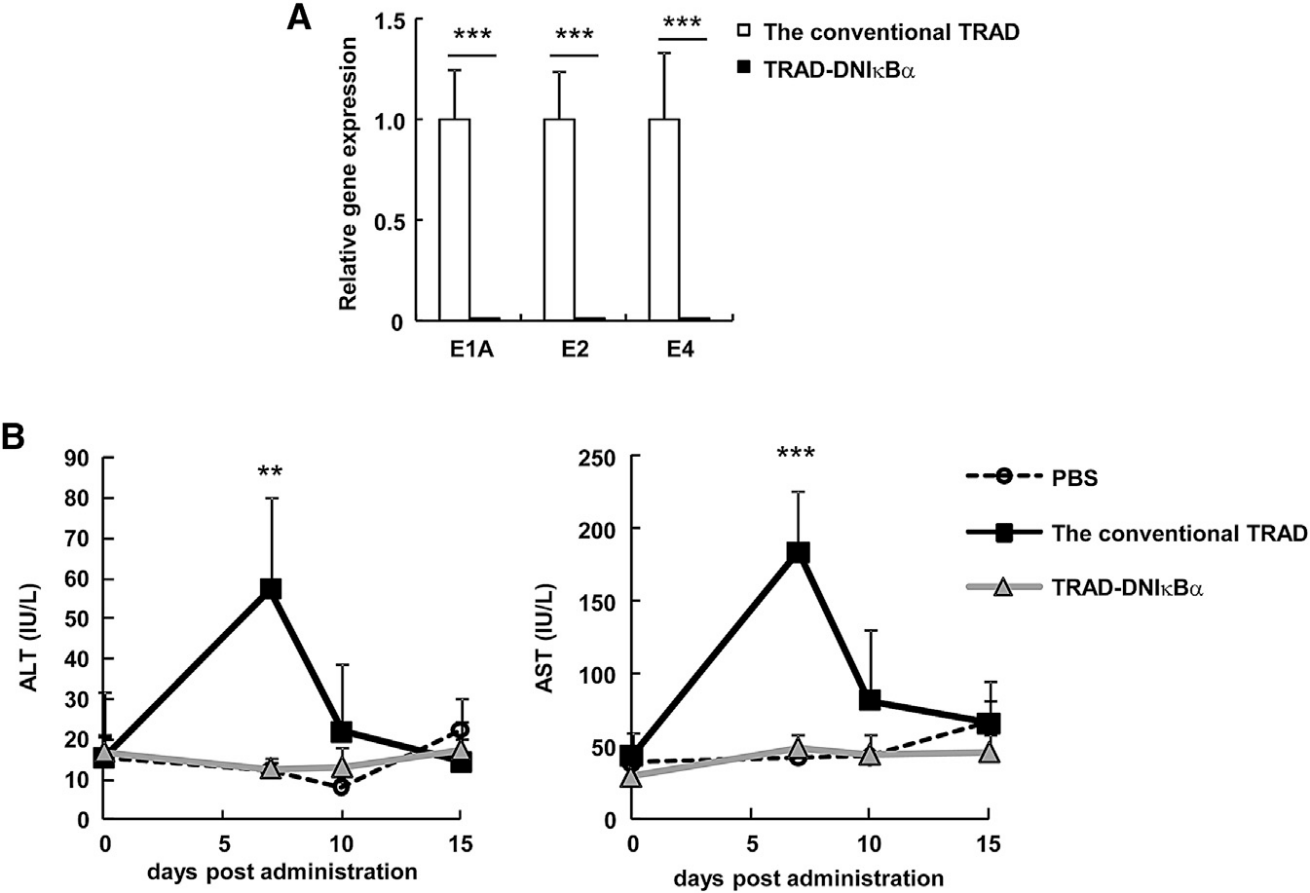
Suppression of TRAD-Mediated Hepatotoxicity in Mouse Liver by the Expression of a Dominant-Negative IκBα (A) C57BL/6 mice were intravenously administered 10^9^ IFU of the conventional TRAD or TRAD-DNIκBα, and 48 hr later, the E1, E2, and E4 mRNA levels in the liver were determined by qRT-PCR analysis. (B) C57BL/6 mice were intravenously administered 10^9^ IFU of the conventional TRAD or TRAD-DNIκBα. At the indicated time points, the serum ALT and AST levels were determined. Data are the means ± SE (n = 5–6). **p < 0.01; ***p < 0.001, TRAD versus TRAD-DNIκBα.

To determine whether the liver-specific inhibition of NF-κB leads to the suppression of TRAD-mediated hepatotoxicity via a reduction in Ad gene expression in the liver, we measured serum alanine aminotransferase (ALT) and aspartate aminotransferase (AST) levels, which are representative biomarkers of hepatotoxicity, after an intravenous administration of the conventional TRAD or TRAD-DNIκBα in mice. Compared with the expression levels of the Ad genes from the conventional TRAD, TRAD-DNIκBα showed more than 90% suppression of the Ad gene expression in the liver (Figure 3A). No apparent increase in serum ALT or AST levels was observed following the administration of TRAD-DNIκBα, whereas the conventional TRAD induced significant elevations in those levels (Figure 3B). These results indicate that TRAD-DNIκBα induces no apparent hepatotoxicity by inhibiting NF-κB signaling and suppressing the leaky expression of Ad genes in the liver.

### Oncolytic Activities of TRAD-DNIκBα against Human Non-hepatic Tumor Cells

**Figure 4:**
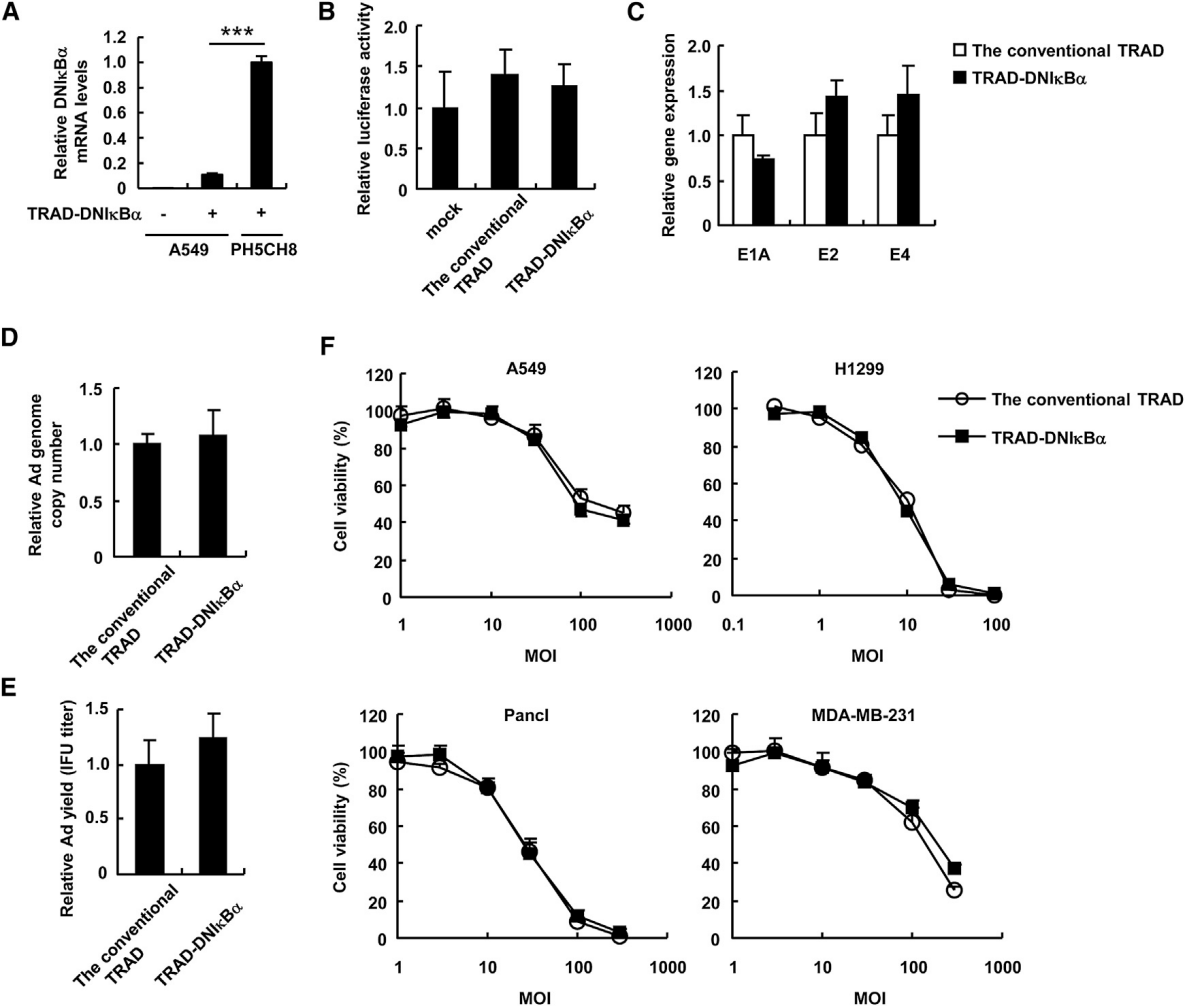
Tumor Cell Lysis Activity of the TRAD Expressing a Dominant-Negative IκBα in Human Non-hepatic Tumor Cells (A) A549 and PH5CH8 cells were infected with TRAD-DNIκBα at an MOI of 5. After 48-hr incubation, the DNIκBα mRNA levels were determined by qRT-PCR analysis. ***p < 0.001. (B) A549 cells were transfected with pNF-κB-Luc, followed by infection with the conventional TRAD or TRAD-DNIκBα at an MOI of 5. After 48-hr incubation, luciferase activity was determined. The data show FLuc activity normalized by RLuc activity. (C–E) A549 cells were infected with the conventional TRAD or TRAD-DNIκBα at an MOI of 5. After 48-hr incubation, the E1A, E2, and E4 mRNA levels (C), the copy numbers of TRAD genomic DNA (D), and the IFU titers of progeny TRAD (E) in the cells were determined by qRT-PCR analysis, qPCR analysis, and infectious titer assay, respectively. (F) A549, H1299, PancI, and MDA-MB-231 cells were infected with the conventional TRAD or TRAD-DNIκBα at the indicated MOIs. After 5-day incubation, cell viability was determined by alamarBlue assay. The data were normalized by the data of the mock-infected group. Data are the means ± SD (n = 4).

To determine whether the inhibition of NF-κB affects oncolytic activities of TRAD against human non-hepatic tumor cells, we infected A549 cells (a human lung adenocarcinoma epithelial cell line) with the conventional TRAD or TRAD-DNIκBα at an MOI of 5. The DNIκBα was slightly expressed in A549 cells following infection with TRAD-DNIκBα, although the DNIκBα expression levels in A549 cells were much lower than those in PH5CH8 cells (Figure 4A). Next, we examined the NF-κB activity after infection with the conventional TRAD or TRAD-DNIκBα. Firefly luciferase (FLuc) expression in mock-transfected cells was almost at negligible levels (data not shown). More than 10^4^-fold higher levels of Fluc expression were observed after transfection with pNF-κB-Luc in A549 cells, compared with those in mock-treated cells, indicating that NF-κB was activated in A549 cells under a normal condition. The conventional TRAD or TRAD-DNIκBα did not significantly activate NF-κB in A549 cells (Figure 4B). The Ad gene expression levels (Figure 4C), the copy numbers of Ad genome (Figure 4D), and the infectious titer units (IFUs) of progeny Ad (Figure 4E) were not significantly lower in A549 cells infected with TRAD-DNIκBα compared with those in the conventional TRAD-infected cells. TRAD-DNIκBα showed efficient cell lysis activities against several human non-hepatic tumor cells at levels similar to the conventional TRAD (Figure 4F). These results indicate that TRAD-DNIκBα does not inhibit NF-κB signaling in human non-hepatic tumor cells, and thus shows efficient replication and oncolytic activities against human non-hepatic tumor cells.

### Reduced Oncolytic Activities of TRAD-DNIκBα against Hepatic Tumor Cells

**Figure 5:**
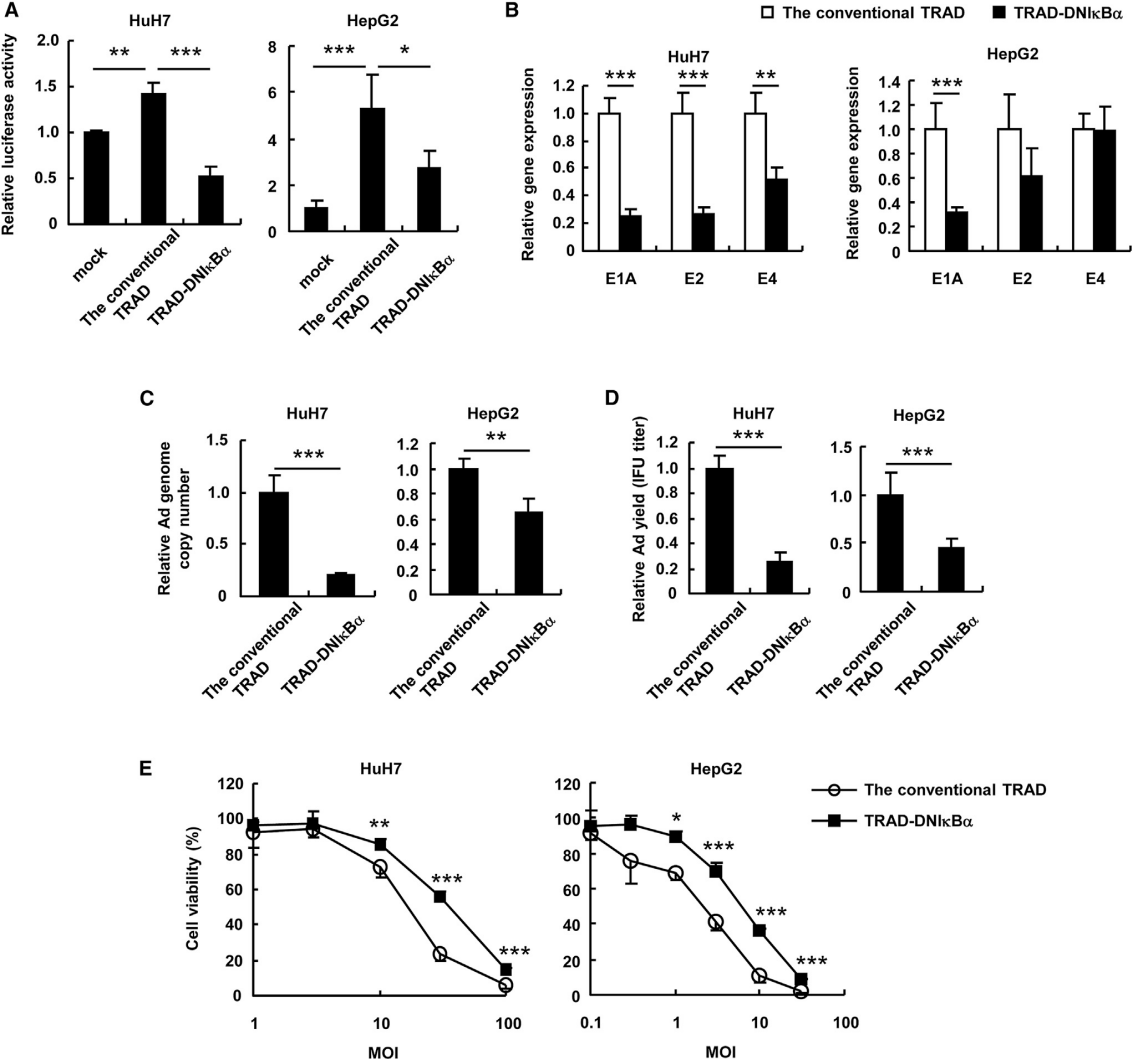
Reduced Tumor Cell Lysis Activity of the TRAD Expressing a Dominant-Negative IκBα in Human Hepatic Tumor Cells (A) HuH7 and HepG2 cells were transfected with pNF-κB-Luc, followed by infection with the conventional TRAD or TRAD-DNIκBα at an MOI of 5. After 48-hr incubation, luciferase activity was determined. The data show FLuc activity normalized by RLuc activity in the cells. (B–D) HuH7 and HepG2 cells were infected with the conventional TRAD or TRAD-DNIκBα at an MOI of 5. After 48-hr incubation, the E1A, E2, and E4 mRNA levels (B), the copy number of TRAD genomic DNA (C), and the IFU titers of progeny TRAD (D) in the cells were determined by qRT-PCR analysis, qPCR analysis, and infectious titer assay, respectively. (E) HuH7 and HepG2 cells were infected with the conventional TRAD or TRAD-DNIκBα at the indicated MOIs. After 5-day incubation, cell viability was determined by alamarBlue assay. The data were normalized by the data of the mock-infected group. Data are the means ± SD (n = 4). *p < 0.05; **p < 0.01; ***p < 0.001.

To determine whether the inhibition of NF-κB affects the oncolytic activities of TRAD against human hepatic tumor cells, we infected HuH7 and HepG2 cells with the conventional TRAD or TRAD-DNIκBα at an MOI of 5. Infection with the conventional TRAD activated NF-κB signaling in HuH7 and HepG2 cells, whereas the activation of NF-κB signaling was significantly suppressed following infection with TRAD-DNIκBα compared with the conventional TRAD (Figure 5A). The Ad gene expression levels (Figure 5B), the copy numbers of Ad genome (Figure 5C), and the IFU titers of progeny Ad (Figure 5D) after infection with TRAD-DNIκBα were significantly lower than those in the conventional TRAD-infected cells. TRAD-DNIκBα showed significantly lower oncolytic activities against HuH7 and HepG2 cells compared with the conventional TRAD (Figure 5E). These results indicate that TRAD-DNIκBα exhibits significant reductions in Ad gene expression and replication in human hepatic tumor cells.

## Discussion

In current cancer virotherapy, intratumoral administration is a main administration route of oncolytic viruses in clinical trials, although a few oncolytic viruses such as reovirus are used via systemic administration.^1^, ^2^ Oncolytic viruses infect and efficiently replicate in tumor cells of injection sites following intratumoral administration. Subsequently, a progeny virus is released from the infected tumor cells and infects the surrounding tumor cells. However, a part of the injected virus and progeny virus is disseminated into the systemic circulation.^28^ In the case of oncolytic Ads, almost all of the disseminated oncolytic Ads accumulate in the liver because of the strong hepatotropism of Ads, leading to the leaky expression of Ad genes from the oncolytic Ads and hepatotoxicity.^9^, ^10^, ^11^, ^12^

In several clinical trials, oncolytic Ads were indeed disseminated from injected tumors and accumulated in the liver.^29^ We reported that Ad gene expression is promoted by NF-κB signaling.^17^ These findings led us to hypothesize that the inhibition of NF-κB signaling results in a suppression of the leaky expression of Ad genes from oncolytic Ads and oncolytic Ad-mediated hepatotoxicity. The aim of the present study was to prevent Ad gene expression and the replication of TRAD in hepatocytes without disturbing oncolytic activities against tumor cells by a hepatocyte-specific expression of DNIκBα. Our present findings demonstrated that TRAD-DNIκBα efficiently inhibited NF-κB signaling in the hepatocytes via a hepatocyte-specific expression of DNIκBα, resulting in the efficient suppression of Ad gene expression from TRAD-DNIκBα and viral replication in the hepatocytes (Figure 2). TRAD-DNIκBα induced no apparent hepatotoxicity following intravenous administration to mice (Figure 3). In addition, TRAD-DNIκBα showed comparable levels of replication efficiencies and oncolytic activities in human non-hepatic tumor cells (Figure 4), whereas oncolytic activities against human hepatic tumor cells were attenuated (Figure 5). TRAD-DNIκBα is not suitable for hepatocarcinoma therapy, although it shows efficient oncolytic activities against non-hepatic cells.

Several modifications have been incorporated into the Ad genome or capsid of oncolytic Ads to prevent oncolytic Ad-induced hepatotoxicity. Cawood et al.^30^ and Sugio et al.^6^ used a microRNA (miRNA)-regulated gene expression system. miRNAs are approximately 22-nt-long noncoding RNAs that bind to the 3′ UTRs of the target gene mRNAs, leading to inhibition of the expression of target genes.^31^ The incorporation of sequences complementary to hepatocyte-specific miR-122a into the E1 expression cassette reduced the replication of oncolytic Ads in a human hepatocyte cell line^30^ and primary human hepatocytes.^6^ In the present study, we suppressed Ad gene expression by a hepatocyte-specific inhibition of NF-κB. TRAD replication was remarkably regulated by the incorporation of a hepatocyte-specific expression cassette of DNIκBα in the hepatocytes (Figure 2). The modification of Ad capsids, such as hexon and fiber proteins, has also been adopted to circumvent oncolytic Ad-mediated hepatotoxicity by regulating the hepatotropism of recombinant Ads. The substitution of Ad fibers derived from Ad serotype 5 with Ad serotype 3 or 35 leads to a significant reduction in the hepatotropism.^32^, ^33^ A modification of Ad capsids with polyethylene glycol also reduced by >90% the hepatotropism after an intravenous administration of Ad.^34^ Combinations of Ad capsid modifications and NF-κB inhibition systems could improve the safety profiles of oncolytic Ads.

Although a variety of strategies for the arming of oncolytic Ads have been adopted to enhance their antitumor activities, these arming strategies might unexpectedly activate NF-κB signaling in hepatocytes. Expression cassettes of inflammatory cytokine genes, including granulocyte macrophage colony-stimulating factor (GM-CSF) and interleukin-12 (IL-12), have been incorporated into the oncolytic virus genome, leading to augmented antitumor immunity.^35^ However, inflammatory cytokines expressed from oncolytic Ads might activate NF-κB signaling in the liver, leading to elevated Ad gene expression and hepatotoxicity. Another system for the regulation of Ad gene expression in normal cells, in addition to a tumor-specific promoter for E1 gene expression in tumor cells, would be highly beneficial to solve these problems.

Our studies, including the present investigation, demonstrated that NF-κB is largely involved in Ad gene expression, and that the inhibition of NF-κB leads to the suppression of Ad-mediated hepatotoxicity.^17^ Several types of viruses, including herpes simplex virus 1 (HSV-1), utilize NF-κB signaling to facilitate viral replication.^18^ HSV-1 possesses NF-κB binding sites in its viral gene promoters, and thus the NF-κB signaling triggers the enhancement of its viral gene expressions, leading to the promotion of the viral replication. HSV-1 infection-induced activation of NF-κB significantly promotes HSV-1 replication via an upregulation of the expression of the viral late proteins, VP16 and gC.^36^, ^37^ Genetically modified HSV-1 has also been developed as an oncolytic virus and showed potent oncolytic activities.^1^, ^2^ An NF-κB inhibition system could also be useful for the control of the replication of an oncolytic HSV-1.

In summary, we have developed an oncolytic Ad showing a hepatocyte-specific expression of DNIκBα (TRAD-DNIκBα). TRAD-DNIκBα showed a significant reduction in Ad gene expression and viral replication in hepatocytes, but not non-hepatic tumor cells, compared with the conventional TRAD. The inhibition of NF-κB in the liver led to a suppression of TRAD-mediated hepatotoxicity. TRAD-DNIκBα offers great potential for safer and effective cancer virotherapy.

## Materials and Methods

### Cells and Mice

PH5CH8 cells (a non-neoplastic human hepatocyte cell line)^20^ were cultured in hepatocyte culture medium (HCM; Lonza, Basel, Switzerland) with 2% fetal bovine serum (FBS). HEK293 (a transformed human embryonic kidney cell line), HuH7 (a human hepatocellular carcinoma cell line; RCB1366; obtained from the JCRB Cell Bank, Tokyo, Japan), HepG2 (a human hepatocellular carcinoma cell line; RCB1648; obtained from the JCRB Cell Bank), A549 (a human lung adenocarcinoma epithelial cell line), PancI (a human pancreatic carcinoma cell line), and MDA-MB-231 (a human breast carcinoma cell line) cells were cultured in DMEM supplemented with 10% FBS, streptomycin (100 μg/mL), and penicillin (100 U/mL). H1299 cells (a human non-small cell lung carcinoma cell line) were cultured in RPMI 1640 supplemented with 10% FBS, streptomycin (100 μg/mL), and penicillin (100 U/mL). Six-week-old female C57BL/6 mice were obtained from Nippon SLC (Shizuoka, Japan). All animal experiments were approved by the Animal Experiment Committee of Osaka University.

### Plasmids

pNF-κB-Luc, which contains the FLuc gene expression cassette driven by a promoter containing a consensus sequence for NF-κB binding, was purchased from Agilent Technologies (Santa Clara, CA, USA). pLuc, which is a control plasmid lacking the consensus sequence for NF-κB binding, was also purchased from Agilent Technologies. pHMCMV-RLuc, a reporter plasmid carrying a cytomegalovirus (CMV) promoter-driven renilla luciferase (RLuc) expression cassette, was previously constructed.^38^

pAdHM23-hAIB-AHADNIkBa, a plasmid for a TRAD carrying an AHA promoter^25^-driven DNIκBα gene expression cassette, was constructed as follows. First, the fragment containing the DNIκBα gene was amplified by PCR using pCMX-IκBαM (Addgene plasmid 12329; Addgene, Cambridge, MA, USA), which contains the DNIκBα gene expression cassette, and the primers DNIκBα-F and DNIκBα-R, and was ligated with the *Not*I/*Xba*I fragment of pHMAHA6.^27^ The resulting plasmid, pHMAHA6-DNIκBα, was digested with BglII and then ligated with BamHI-digested pHM15,^39^ resulting in pHM15-AHA-DNIκBα.

Next, an *Xba*I recognition site was introduced into the E3-deleted region of pAdHM4,^40^ an Ad plasmid encoding the *Pac*I-flanked Ad vector genome that lacks the E1 (bp 342–3,523) and E3 (bp 28,133–30,818) regions. The resulting plasmid, pAdHM23-XbaI, was digested with *Xba*I and then ligated with *Spe*I-digested pHM15-AHA-DNIκBα, resulting in pAdHM23-AHA-DNIκBα. Next, pHM5-hAIB,^6^ in which the E1A and E1B genes linked with an internal ribosomal entry site (IRES) are located downstream of an hTERT promoter, was digested with I-*Ceu*I/PI-*Sce*I and then ligated with I-*Ceu*I/PI-*Sce*I-digested pAdHM23-AHA-DNIκBα. Further details on the construction methods are available upon request.

### Oncolytic Ads

Recombinant Ads were prepared as follows. *Pac*I-digested pAdHM19-hAIB^41^ and pAdHM23-AHA-DNIκBα were transfected into HEK293 cells using Lipofectamine 2000 (Life Technologies, Carlsbad, CA, USA), resulting in the production of the conventional TRAD and TRAD-DNIκBα, respectively. These TRADs were amplified and purified by two rounds of cesium chloride-gradient ultracentrifugation, dialyzed, and stored at −80°C.^40^ The IFU titer was measured by serial titration on HEK293 cells, followed by determination of the numbers of Ad-infected cells using an Adeno-X Rapid Titer Kit (Clontech, Mountain View, CA, USA). The ratio of the particle to biological titer was between 5.7 and 9.1 for each Ad vector used in this study.

### Cell Viability and Cytotoxicity Assay

For the evaluation of the effects of NF-κB inhibitors on cell viability, we treated HuH7 cells with BAY11-7082 and MG-132 (Invivogen, San Diego, CA, USA). After 24-hr incubation, the cell viabilities were determined by staining with alamarBlue (Life Technologies) according to the manufacturer’s instructions.

For the evaluation of the cell lysis activities of TRADs, cells were infected with TRADs at the indicated MOIs. After 5-day incubation, cell viabilities were determined as described above.

### Analysis of NF-κB Activities Using Reporter Plasmids

Cells were co-transfected with pLuc or pNF-κB-Luc (500 ng/mL) and pHMCMV-RLuc (160 ng/mL) using Lipofectamine 2000 (Life Technologies). Following 6-hr incubation, the cells were infected with TRADs. After a total 48-hr incubation, luciferase activities in the cells were determined using the Dual Luciferase Reporter Assay System (Promega, Madison, WI, USA).

For the evaluation of the effects of NF-κB inhibitors on the NF-κB signaling, cells were treated with BAY11-7082 and MG-132 (Invivogen) 24 hr after transfection with plasmids as described above. A luciferase assay was performed 24 hr after the NF-κB inhibitor treatment.

### qRT-PCR Analysis

Cells were infected with TRADs at the indicated MOIs. Following the indicated incubation periods, total RNA was isolated from the cells using ISOGEN (Nippon Gene, Tokyo). Next, cDNA was synthesized using 500 ng of total RNA with a Superscript VILO cDNA synthesis kit (Life Technologies). A qRT-PCR analysis was performed using Fast SYBR Green Master Mix (Life Technologies) and a StepOnePlus real-time PCR system (Life Technologies) as described previously.^42^ The sequences of the primers used in this study are provided in Table S1.

For the inhibition of NF-κB, cells were pretreated with BAY11-7082 and MG-132 at 10 and 2.5 μM, respectively, for 1 hr, followed by infection with TRADs. Total RNA was recovered 24 hr after the addition of TRADs, followed by an Ad gene expression analysis as described above.

### Determination of Ad Genome Copy Numbers and Infectious Titers in the Cells

Cells were treated with TRADs using a protocol similar to that described above. Following the indicated incubation periods, total DNA, including Ad genomic DNA, was isolated from the cells infected with Ads, using a DNeasy Blood & Tissue Kit (QIAGEN, Hilden, Germany). After isolation, the Ad genome copy numbers were quantified by qPCR analysis using the StepOnePlus real-time PCR system as described previously.^43^ The sequences of the primers and probes used in this study are provided in Table S1.

For the determination of infectious titers following infection with TRADs, cells were recovered and subjected to three cycles of freezing and thawing. After centrifugation, the supernatants were added to HEK293 cells. After incubation for 48 or 72 hr, we analyzed the numbers of cells infected with Ads by using an Adeno-X Rapid Titer Kit (Clontech).

### Analysis of Ad Gene Expression in the Liver and Hepatotoxicity following TRAD Administration in Mice

TRADs were intravenously administered to mice at a dose of 10^9^ IFUs/mouse via the tail vein. Total RNA was extracted from the livers 48 hr after administration, and the Ad gene mRNA levels were determined by qRT-PCR analysis. The blood samples were collected via retro-orbital bleeding at the indicated days, followed by centrifugation to recover the serum samples. The serum ALT and AST levels were determined using a transaminase-CII-test kit (Wako, Osaka, Japan).

### Statistical Analysis

Statistical significance was determined using Student’s t test. Data are presented as the means ± SD or SE.

## Author Contributions

M.M. designed and performed the experiments, analyzed data, and wrote the manuscript. F.S. designed and supervised the projects, analyzed data, and wrote the manuscript. K.W. and K.N. supported the experiments. M.T. analyzed data. N.K. and T.F. provided the materials and supported the experiments. H.M. supervised the projects, interpreted data, and wrote the manuscript.

## Conflicts of Interest

T.F. and H.M. are consultants of Oncolys BioPharm, Inc. No potential conflicts of interest were disclosed by the other authors.

## Supplemental Information

Document S1. Figures S1–S3 and Table S1

Document S2. Article plus Supplemental Information
