# Supplementary material for: Suppression of Oncolytic Adenovirus-Mediated Hepatotoxicity by Liver-Specific Inhibition of NF-κB
Source: Mol Ther Oncolytics. 2017 Oct 26;7:76–85. doi: 10.1016/j.omto.2017.10.003 (PMC5704103; doi:10.1016/j.omto.2017.10.003)
Supplement: Document S1. Figures S1–S3 and Table S1 [file mmc1.pdf]

OMTO, Volume 7

Supplemental Information

Suppression of Oncolytic Adenovirus-Mediated Hepatotoxicity by Liver-Specific Inhibition of NF-κB

Mitsuhiro Machitani, Fuminori Sakurai, Keisaku Wakabayashi, Kosuke Nakatani, Masashi Tachibana, Nobuyuki Kato, Toshiyoshi Fujiwara, and Hiroyuki Mizuguchi

Supplemental Figures

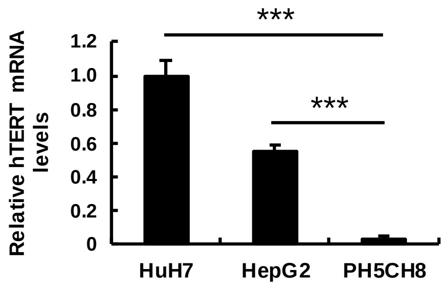

Suppl. Fig. S1. Expression levels of human telomerase reverse transcriptase (hTERT) in PH5CH8 cells. hTERT mRNA levels were determined by qRT-PCR analysis. The ratios of hTERT to GAPDH mRNA levels are shown. The data of HuH7 cells was normalized to 1. Data are the means±SD (n=4). ***p<0.001.

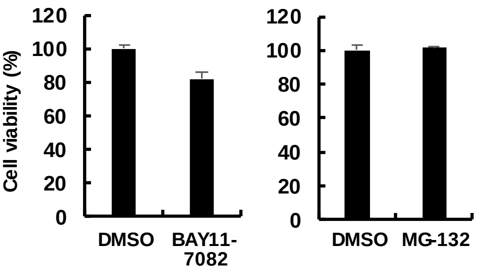

Suppl. Fig. S2. Viability of PH5CH8 cells after treatment with NF-κB inhibitors. PH5CH8 cells were treated with BAY11-7082 and MG-132 at 5 μM and 2.5 μM, respectively. After 24-hr incubation, cell viability was determined by alamarBlue® assay. Data are the means±SD (n=4).

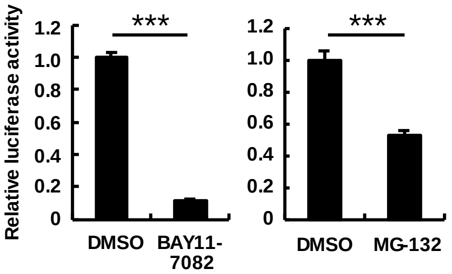

Suppl. Fig. S3. NF-κB activity in PH5CH8 cells after treatment with NF-κB inhibitors. PH5CH8 cells were pretreated with BAY11-7082 and MG-132 at 5 μM and 2.5 μM, respectively for 1 hr, followed by transfection with pNF-κB-Luc. After 24-hr incubation, luciferase activity was determined. The data show FLuc activity normalized by RLuc activity in the cells. Data are the means±SD (n=4).

Suppl. Table S1
The primers used in this study

No	name	sequence
1	DNIkBa-F	tgtctagacagccatgtttcagccagc
2	DNIkBa-R	aagcggccgcttataatgtcagacgctggcc
3	E1A-F	tccggtccttctaacacacctc
4	E1A-R	acggcaactggtttaatggg
5	E2-F	cactacggtgcgagtgcaa
6	E2-R	ggtagctgccttcccaaaaag
7	E4-F	gggatcgtctacctccttttga
8	E4-R	gggcagcagcggatgat
9	hGAPDH-F	ggtggtctcctctgacttcaaca
10	hGAPDH-R	gtggtcgttgagggcaatg
11	mGAPDH-F	caatgtgtccgtcgtggatct
12	mGAPDH-R	gtcctcagtgtagcccaagatg
